# Occurrence of erythemato-purpuric patches upon contact with a myriapod (diplopod)

**DOI:** 10.1590/0037-8682-0552-2021

**Published:** 2021-12-17

**Authors:** Sergio de Almeida Basano, Cipriano Ferreira da Silva, Dionatas Ulises de Oliveira Meneguetti

**Affiliations:** 1 Centro de Pesquisa em Medicina Tropical de Rondônia, Porto Velho, RO, Brasil.; 2 Secretaria de Saúde do Estado de Rondônia, Porto Velho, RO, Brasil.; 3 Universidade Federal de Rondônia, Porto Velho, RO, Brasil.; 4 Universidade Federal do Acre, Programa de Pós-Graduação Stricto Sensu em Ciências da Saúde na Amazônia Ocidental, Rio Branco, AC, Brasil.; 5 Universidade Federal do Acre, Programa de Pós-Graduação Stricto Sensu em Ciência, Inovação e Tecnologia para Amazônia, Rio Branco, AC, Brasil.; 6 Universidade Federal do Acre, Colégio de Aplicação, Rio Branco, AC, Brasil.

A 40-year-old woman presented erythematous patches that evolved with hyperchromia on her left forearm after contact with a myriapod (Figure 1). She complained of a local burning sensation for the past 48 hours, and two mirrored erythemato-purpuric patches of linear configuration were present in the left antecubital fold. We noticed the occurrence of marked epidermal necrosis obscuring exogenous pigmentation, which is cited as the primary pathogenic mechanism. When pressed or crushed, diplopods tend to release chemical substances, such as quinones and hydrogen cyanide, that induce an erythemato-purpuric inflammatory process followed by prolonged residual hypo- and/or hyperpigmentation^1^. Hypopigmentation results from temporary intervention of the functional activity of the epidermo-melanic unit presenting increased melanin production at the epidermis basal layer, while confetti hyperpigmentation results from follicular melanin activity. The follicular unit constitutes adnexal reserve during regenerative processes in the skin generating hyperchromic spots of follicular size and density. These macules are usually serpiginous or rounded based on the diplopod’s anatomical configuration. The tissue damage is proportional to the toxin’s nature and volume and exposure time^2^. During rapid exposure, only exogenous pigmentation may appear. However, extreme cases carry the risk of blister formation, ulceration, and epidermal necrosis^2^. In this case, the inflammatory response induced epidermal necrosis, which was assessed using erythemato-purpuric staining, followed by desquamation with achromia on the topography of the damage, reactionary erythema, and perifollicular hyperchromia (Figure 1). Such spots usually disappear after a few weeks or months without scarring^3^. 

**FIGURE 1:**
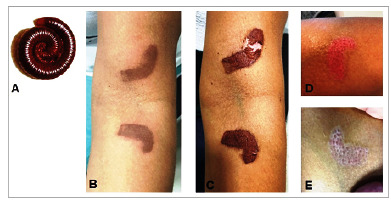
Lesion caused by a myriapod (diplopod). **(A)** Myriapod (diplopod) that caused the lesion; **(B)** Erythemato-purpuric stained lesion; **(C)** Desquamation with achromia atop the damage; **(D)** Reactive erythema; and **(E)** Perifollicular hyperchromia.

## ETHICAL COMMENTS

The patient signed an informed consent form. Since this is a case report, ethics committee approval was not required.

## References

[1] Haddad V, Manço DG (2019). An unusual dark macular lesion in the plantar region of a child. Rev Soc Bras Med Trop.

[2] Radford AJ (1975). Millipede burns in man. Trop Geogr Med.

[3] Fracaroli TS, Miranda LQ, Maceira JP, Barcaui CB (2015). Exogenous pigmentation after Diplopoda exposure leading to a dermatoscopic parallel ridge pattern on the plantar region. J Dermatol Case Rep.

